# Selecting reference genes in RT-qPCR based on equivalence tests: a network based approach

**DOI:** 10.1038/s41598-019-52217-2

**Published:** 2019-11-07

**Authors:** Emmanuel Curis, Calypso Nepost, Diane Grillault Laroche, Cindie Courtin, Jean-Louis Laplanche, Bruno Etain, Cynthia Marie-Claire

**Affiliations:** 1 UMR-S 1144, INSERM Optimisation thérapeutique en neuropsychopharmacologie – université Paris Descartes – université Paris Diderot, Paris, France; 2 Laboratoire de biomathématiques, EA 7537 BioSTM, faculté de pharmacie de Paris, université Paris Descartes, Paris, France; 3 Service de biostatistiques et d’informatique médicale, hôpital Saint-Louis, AP-HP, Paris, France; 4 AP-HP, GH Saint-Louis – Lariboisière – F. Widal, Pôle de psychiatrie et de médecine addictologique, 75475 Paris, cedex 10 France; 5 Fondation Fondamental, Créteil, France

**Keywords:** Reverse transcription polymerase chain reaction, Statistical methods

## Abstract

Because quantitative reverse transcription PCR (RT-qPCR) gene expression data are compositional, amounts of quantified RNAs must be normalized using reference genes. However, the two most used methods to select reference genes (NormFinder and geNorm) ignore the compositional nature of RT-qPCR data, and often lead to different results making reliable reference genes selection difficult. We propose a method, based on all pairwise equivalence tests on ratio of gene expressions, to select genes that are stable enough to be used as reference genes among a set a candidate genes. This statistical procedure controls the error of selecting an inappropriate gene. Application to 30 candidate reference genes commonly used in human studies, assessed by RT-qPCR in RNA samples from lymphoblastoid cell lines of 14 control subjects and 26 patients with bipolar disorder, allowed to select 7 reference genes. This selection was consistent with geNorm’s ranking, less with NormFinder’s ranking. Our results provide an important fundamental basis for reference genes identification using sound statistics taking into account the compositional nature of RT-qPCR data. The method, implemented in the SARP.compo package for R (available on the CRAN), can be used more generally to prove that a set of genes shares a common expression pattern.

## Introduction

Comparison of gene expression levels among biological samples is used in a wide range of experimental conditions. The most popular method to quantify gene expression is quantitative reverse transcription PCR (RT-qPCR). This method offers several advantages: high sensitivity, relatively low cost of reagents and high adaptability to a wide range of experimental designs. However, like in most experiments performed to quantify the amounts of RNAs present in a sample, the *total* amount of RNA input is fixed in RT-qPCR. Because of this constraint, any change in the amount of a single RNA will necessarily translate into opposite changes on all other RNA levels i.e. the RNA amounts are compositional, and their sum equals a fixed amount. This implies that interpreting changes of one single gene expression without reference is impossible.

To overcome this limitation, in experiments designed to study the differential expression of a set of candidate genes between two, or more, conditions, “reference genes” (also known as control genes or housekeeping genes) are used to normalize the amounts of quantified RNAs. A reference gene is a gene whose expression is stable in all studied conditions. It would ideally have exactly the same expression level in all samples, with only measurement noise added. In practice, it is expected to have a very low variability of expression in each of the tested conditions, and an equal average level of expression between all tested conditions. Normalization using a single reference gene is risky as stated in the MIQE (Minimum Information for Publication of Quantitative Real-Time PCR Experiments) guidelines^1^. In addition, appropriate reference genes should not belong to the same pathway or the same gene family. We have recently published a method that allows to relax the assumptions on reference genes^2^. However, using reference genes, in a given experiment, might still help the interpretation of the results.

Hence, selection of reference genes is a key procedure in the design of RT-qPCR experiments, especially in differential expression experiments. Such a selection assumes that one can assess that the expression level of a given reference gene does not change between the studied conditions. However, this is an impossible task with RT-qPCR data because of their compositional nature. To overcome this limitation, the underlying idea is that gene expressions that do change will not change all identically. Consequently, a large set of genes, which behave similarly, probably corresponds to genes whose expression is not modified between the conditions – an external information that is mandatory to allow interpretation of compositional data at the individual gene level.

Several methods have been proposed to select sets of genes that experience very similar changes between different experimental conditions. The two most frequently used are the geNorm^3^ and the NormFinder^4^ methods. Both methods rank the candidate reference genes by order of increased stability. However, there is no objective criterion to select the “best” reference genes in this ranking since there is no guarantee that the “best” gene, according to this ranking, is stable enough to be used as a reference gene. There is no guarantee either that the “worst” gene is indeed unusable, since ranking could be the simple reflect of experimental variability. Besides, the two methods often lead to different rankings^5–10^, with presently no clear justification to prefer one ranking or another.

This paper presents a new method that overcomes these limitations, ensuring that selected genes will be stable enough to be used as reference genes with a statistical procedure that controls the error of selecting an inappropriate gene. This procedure uses an equivalence test to prove the hypothesis that a given pair of genes experiences the same expression change in two different conditions. Haller *et al*.^11^ give a detailed explanation on equivalence tests applied to reference gene selection. Briefly, an equivalence test is a statistical test for the null hypothesis that the unsigned difference between two values is higher than a predefined threshold, Δ^12,13^. Hence, rejecting the null hypothesis allows concluding that the difference is smaller than Δ. It is typically used in bioequivalence studies to demonstrate that a generic treatment has the same properties than its princeps^14,15^. The null hypothesis is rejected with a Type I error lower than 5% if the 90% confidence interval of the mean difference is completely included in the equivalence region, [−Δ, +Δ]. This occurs if both the real change is small, so that the confidence interval is centered on a value in this equivalence region, and the variability is small, so that the confidence interval is narrow enough to completely fit in the equivalence region^16^.

However, because of the compositional nature of the RT-qPCR data, equivalence tests (just as usual difference tests) cannot be performed on data for a single gene. To overcome this difficulty, the equivalence approach was coupled with the approach based on all pairwise ratios and subgraphs analysis previously developed^2^. Briefly, instead of considering a single candidate control gene, the ratio of two candidate genes expressions is considered. The equivalence of this ratio is then tested between the different experimental conditions. If the ratio does not change (the test is significant), both candidate genes share the same expression change between the conditions. The mathematical details of the underlying model are given in Supplementary Material. It should be stressed that, because of the compositional nature of the RT-qPCR data, no method can go further without using external information. All possible ratios are tested for equivalence, and a graph is built based on the following rules: (1) each node of the graph is one of the candidate genes; (2) two nodes are linked if, and only if, the equivalence test is significant. Last, the biggest subset of nodes that are all connected together (a “maximal clique” in graph theory) is selected as the set of reference genes. If several such cliques exist, their intersection defines this set.

To test the method, a set of 30 candidate reference genes commonly used in human studies was evaluated in lymphoblastoid cell lines (LCLs) from healthy volunteers and patients with bipolar disorder. The selected set of reference genes was compared to the ranking obtained using the geNorm and NormFinder methods. Results were also compared to the individual genes experimental variability, either intra-assay variability or inter-individual variability in a given group of patients. Another example with 5 candidate reference genes is fully detailed as Supplementary File, as a tutorial on how to apply this method for routine labwork, along with an example R code.

## Results

### Intra-assay variability

**Figure 1:**
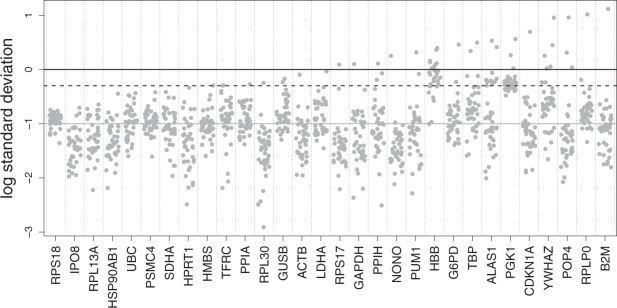
Standard deviations of the three technical replicates for all samples, all genes (logarithmic scale). Each dot is the standard deviation of a given sample for a given gene. Black line: 1 Cq; black dashed line: 0.5 Cq; grey continuous line: 0.1Cq. Genes are ordered by increasing value of the maximal standard deviation observed amongst all samples.

Intra-assay (analytical) variability was defined as the standard deviation of the three technical replicates, for all genes and all patients. Results are presented on Fig. 1. Sixteen technical replicates failed, associated with five genes (*G6PD* [1 failure], *HBB* [10 failures, including one complete sample], *POP4* [2 failures, on the same sample], *RPLP0* [1 failure], and *RPS17* [2 failures, on different samples]). Such genes were considered as second choice reference genes, since such failures may prevent further data analysis; especially, gene *HBB* with 62.5% of all failures was considered as unusable as a reference gene. It was checked that the proposed method did not suggest this gene in the final selection.

Figure 1 suggests that analytical variability was around 0.1 on the Cq scale at best, meaning that equivalence regions below this value (using Δ < 0.1) are unrealistic. It also illustrates that *HBB* and *PGK1* genes have a much higher analytical variability than all others; consequently, the proposed method should not select these genes as reference genes. This higher analytical variability is likely related to lower RNAs amounts in the sample, translating into higher Cq; indeed, analytical variability increases with Cq, as expected (Supplementary Fig. 1).

A few triplicates presented an abnormally high standard deviation (more than 1, and up to 13 for *B2M* in one patient). Most of these large standard deviations were tracked down to large outliers; in agreement with standard practice, such outliers were removed before technical replicates average. All further analyses were done on these averaged Cq.

### Expression levels and variation in reference genes

**Figure 2:**
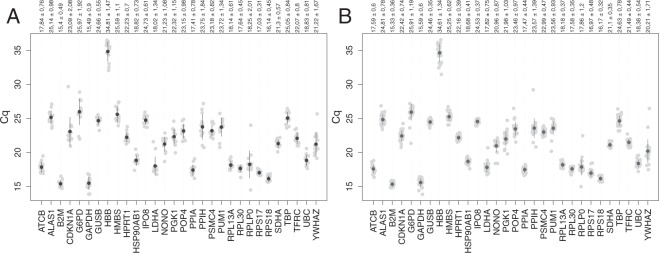
Cq value (average of technical replicates) of each sample from control (**A**) or patients (**B**), for each gene. Grey dots are the individual values; black dot is the mean Cq for the given gene; segments are mean ± standard deviation. Genes are sorted by alphabetical order. Values above are mean ± standard deviation.

The distribution of Cq values (mean of triplicates) for all genes and samples is shown in Fig. 2. The Cq values ranged from 13.85 (*GAPDH*) to 36.54 (*HBB*) in patients and from 13.87 (*GAPDH*) to 36.48 (*HBB*) in controls. *B2M* and *HBB* have the lowest (15.33 in patients) and highest (34.81 in controls) Cq value, respectively. In the control samples, *RPLP0* shows the highest variable expression level with Cq values ranging from 14.49 to 22.45 while in the patients’ samples *YWHAZ* showed a large range of expression (17.47 to 26.45). The gene showing the smallest expression level range was *RPS17* (16.62 to 17.65) in control samples and *RPS18* (15.62 to 16.85) in samples from bipolar patients. When considering all samples, the Cq values of *RPS18* and *RPLP0* exhibited the smallest (15.62 to 16.85) and the largest expression ranges (14.49 to 23.99), respectively. These results show that the expression levels of the 30 reference genes tested in LCLs from bipolar patients and control subjects varied significantly. We then screened for the best reference genes under these specific experimental conditions.

The inter-individual variability of the Cqs, in a given group, ranged from 0.31 (*RPS17*, controls) to 2.08 (*CDKN1A*, controls). This result suggests that equivalence regions narrower than ±0.3 will hardly detect reference genes and, conversely, that *CDKN1A* is not an acceptable reference gene because of its high inter-individual variability.

### Expression stability levels of reference genes using the equivalence tests procedure

**Figure 3:**
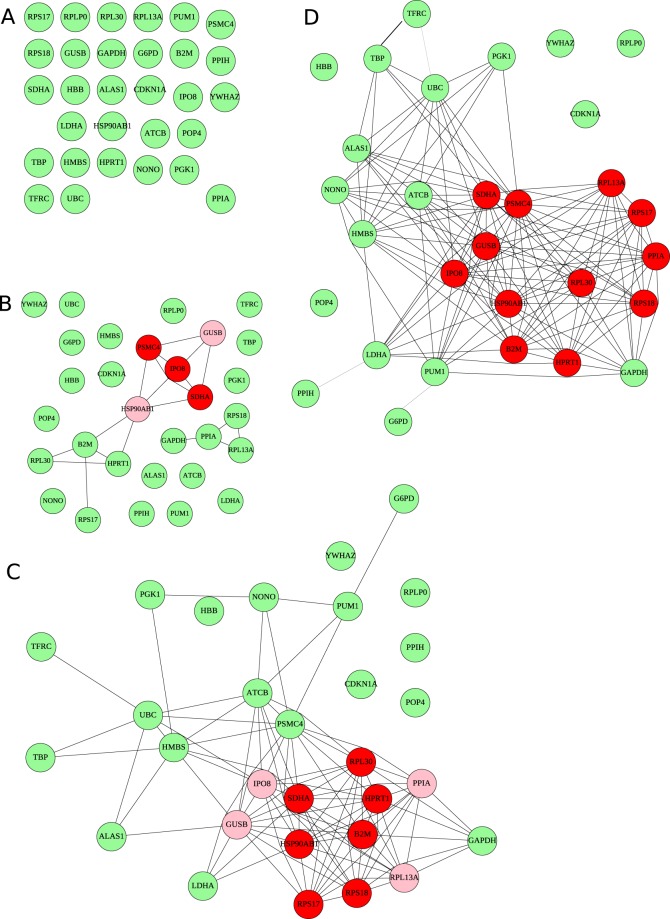
Equivalence graphs obtained using Δ = 0.1 (**A**), Δ = 0.3 (**B**), Δ = 0.5 (**C**) and Δ = 0.6 (**D**). Each node is a candidate gene. Connected nodes are equivalent according to the equivalence test for the corresponding [−Δ; +Δ] equivalence region, at *p* < 0.3 (see text). Nodes with red background belong to all maximal cliques of the graph. Nodes with pink background belong to at least one maximal clique. Other nodes, with green background, do not belong to any maximal clique, hence are not selected as good reference genes.

For 30 genes, the cut-off for individual equivalence tests was *p* < 0.3 – that is; an edge between the nodes representing two genes is added if the equivalence test is significant with *p* < 0.3 – to ensure that the probability of seeing maximal cliques of more than two nodes is less than 0.05 if no such clique exists. The resulting graph is presented in Fig. 3C for the predefined equivalence region of [−0.5; +0.5]. Results with other equivalence regions are shown in Fig. 3A (Δ = 0.1, typical analytical variability), 3B (Δ = 0.3, inter-individual intra-group lowest variability), and 3D (Δ = 0.6). As expected, the larger Δ, the less stringent the constraint on what is called “similar variations”, and the more equivalent genes are detected. For the most stringent Δ = 0.1 equivalence region, there is not a single connection: there is no pair of gene that has a low enough variability to prove that their differential expression change between controls and patients is smaller than 0.1. For the Δ = 0.3 equivalence region, two groups of connected nodes appear; the two maximal cliques contain 4 nodes and share three nodes: *IPO8*, *PSMC4*, and *SDHA*. For the predefined Δ = 0.5 equivalence region, there is a single connected subgraph, which includes 3 maximal cliques of 9 nodes each; their intersection contains 7 genes *B2M*, *HPRT1*, *HSP90AB1*, *RPL30*, *RPS17*, *RPS18*, and *SDHA*. All of these 7 genes are mutually equivalent, at the specified equivalence threshold, and are the most promising candidates as reference genes.

Even with the less stringent equivalence region, the single maximal clique contains only 12 of the 30 genes (the seven above, *GUSB*, *IPO8*, *PPIA*, *PSMC4*, and *RPL13A*). In addition, five genes (*POP4*, *HBB*, *YWHAZ*, *RPLP0*, and *CDKN1A*) appear isolated: they could not be associated with any other gene. This could result either from a regulation of their expression different from all other tested genes, or from a too high analytical or inter-individual variability. In both cases, these genes should be excluded as reference genes. Of note, amongst them are some of the genes excluded either for their large intra-assay (*HBB*) or inter-individual variability (*CDKN1A*). As shown in Figs 1 and 2, all of these five genes had high intra-assay variability; *POP4*, *RPLP0* and *YWHAZ* also present numerous outliers. This suggests that variability issues are the main reason for rejecting these genes as reference genes.

### Comparison with geNorm and NormFinder

**Figure 4:**
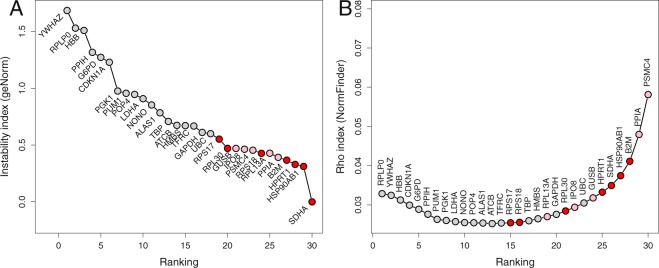
Comparison of candidate genes selection by the equivalence approach and the ranking given by the geNorm (**A**) and the NormFinder (**B**) methods. Genes are ranked in increasing stability from left to right. Reds dots are for genes selected (maximal cliques) by the equivalence method with Δ = 0.5; pink dots, for genes selected with Δ = 0.6.

The expression stability of the candidate genes was also analyzed using geNorm and NormFinder methods. The two methods give a different ranking (Fig. 4), with the best three genes considered by geNorm (*SDHA*, *HSP90AB1*, *HPRT1*) being classified as candidates 5, 4, and 6 respectively by NormFinder; conversely, the best three genes according to NormFinder (*PSMC4*, *PPIA*, *B2M*) are classified as candidates 8, 5, and 4 respectively by geNorm. Regarding the comparison with the equivalence test procedure, the results also differ between geNorm and NormFinder, but the 7 best candidates include 4 (NormFinder) or 5 (geNorm) of our candidates. Of note, with Δ = 0.6, the 12 genes of the maximal clique are also the 12 best candidates according to the geNorm algorithm. The situation is not so clear for the NormFinder algorithm with the most stable genes according to the equivalence test procedure being scattered in the NormFinder ranking list, with *RPS17* being proposed at the 15^th^ position only.

## Discussion

Identification of suitable candidate reference genes is crucial for RT-qPCR analyses of gene expression. Several methods have been developed but they ignore the compositional nature of RT-qPCR data. We propose here an equivalence test procedure to select appropriate reference genes and illustrate it on a set of 30 reference genes commonly used in human studies and for which primers are commercially available. Detailed steps of the procedure are presented, on another dataset, in Supplementary Materials along with R code.

The proposed equivalence test procedure has several advantages compared on other methods. First, it takes into account the compositional nature of RT-qPCR data. Due to the fixed amount of RNA input in the experiment any change in a given RNA amount modify the amount of all the other RNAs^2^. This problematic issue is overcome by testing for equivalence all possible ratios between pairs of candidate reference genes and linking together genes that behave similarly. Second, this method provides an easy to interpret result. In the built graph, the biggest subset of genes that are all connected together identifies the preferential set of reference genes. Another advantage is that this method clearly defines a threshold of acceptable expression stability. Obviously, more stringent this threshold is, less genes will be present in the final maximal clique. The example in this study is performed with Δ = 0.5. By varying this Δ value, genes will be added (if Δ is increased) or excluded (if Δ is decreased) from the candidate set. The order of genes exclusion could be used as a ranking of the genes. This ranking could however be partial, because the set could also be split in subparts of equal or unequal number of nodes, each part having more than one node.

Therefore, one of the difficulties is the minimal number of genes to be included in a clique in order to flag it as a potential clique of reference genes. Obviously, fixing a *p* < 0.3 threshold, individual edges between two nodes can appear randomly quite frequently, hence pairs of genes are not reliable indications of stable genes. However, the probability of having maximal cliques of increasing size vanishes quickly with randomly selected edges, since a maximal clique of size N implies that all N(N − 1)/2 edges are present – 3 edges for 3 nodes, 6 for 4 nodes and so on. Hence, we suggest that maximal cliques of 4 or more candidate genes are reliable. This number depends on the edge creation threshold, which itself increases with the number of candidates. Hence, if only a few candidate genes are studied, the threshold will be lower than 0.3, and lower sized cliques may be used reliably. Hence, this method was used with an independent dataset of LCLs from 33 patients with bipolar disorder. In this dataset, five candidate reference genes were tested and the method identified a maximal clique of three reference genes appropriate to analyze gene expression modulations when comparing male and female patients (Supplementary Materials).

Compared with other methods, our suggestion takes a different approach: instead of ranking genes, it tries to find sets of genes whose expression is equivalent between conditions. This allows first to objectivize what “no expression change” means, by defining the equivalence region (in the Cq scale), and second to alleviate the need of finding a cutoff in the ranking. However, one should expect that the sets found include the top genes ranked by other methods. Indeed, our method gives results that are, at least on this dataset, quite consistent with the geNorm ranking, less consistent with the NormFinder one. Interestingly, in the second dataset, the results obtained with the equivalence test method are in accordance with those obtained with both geNorm and Normfinder (Supplementary Materials). To understand this, the mathematical models underlying the three methods are given in Supplementary Materials, and compared. Briefly, the model underlying the NormFinder algorithm partially accounts for the compositional nature of the data, and assumes a contradictory hypothesis of centered Gaussian distribution for a parameter that should always be negative (see Supplementary Materials). This may explain its difficulty to detect some candidates whose expression change is smallest than the selected threshold [–Δ, + Δ]. Compared to geNorm, our method offers a natural and objective way to select a set of reference genes, including proving that some of the candidate genes are indeed reference genes, and not only ranking them. Comparison with analytical and intra-group, inter-individual variabilities confirms that our method selects preferentially less variable candidate genes. Due to the compositional nature of RT-qPCR data, one however cannot asset that the change of these candidate genes between groups is indeed negligible, only that all these genes experience similar change of expression.

In the two examples used in this paper, equivalence test was performed based on the assumption that Cq are normally distributed. This assumption is not mandatory for the method: non-parametric equivalence tests exist (see Haller *et al*.^11^ for a discussion about this issue) and could be used instead, as far as a *p*-value can be obtained. Furthermore, we stress that departure from normality in the Cq scale are most often due to asymmetric distributions, heavy tailed distributions or outliers. In each of these cases, the standard deviation will be increased, leading to wider confidence intervals that will difficulty fit in the equivalence region: this makes equivalence tests conservative when assuming a Gaussian distribution. This is even more true in case of outliers, that will additionally shift the mean farther from 0, hence outside the equivalence region, as observed on non-connected nodes when Δ is large in our example.

## Conclusion

This is, to our knowledge, the first study using equivalence tests to identify a set of reference genes for RT-qPCR expression studies. Application to gene expression in LCLs from control subjects and patients with bipolar disorder suggests that it can successfully select genes with the lowest expression variability. Comparisons show consistency with the geNorm method, and discrepancies with the NormFinder method. Our results provide an important fundamental basis for reference genes identification using sound statistics and taking into account the compositional nature of RT-qPCR data. The method is implemented in the SARP compo package for R (starting at version 0.1.0), available on the Comprehensive R Archive Network^17^. Beside reference gene identification, this method can be used to analyze differential expression data aiming to prove that a set of gene shares the same change in expression pattern − that is, a set of coexpressed genes.

## Material and Methods

### Population

The sample consisted of 26 patients with a diagnosis of bipolar disorder according to DSM-IV criteria and 14 controls from the previously described multicentric cohort GAN (Genetic Actigraphy and Neuropsychology, Clinical Trials Number NCT02627404)^18^. Details on the inclusion and exclusion criteria have been described previously^18^. Non-psychiatric controls subjects were recruited as volunteers and through a direct interview were screened for the absence of individual and family history of psychiatric illness. The study was approved by the Comité de protection des personnes from La Pitié-Salpétrière hospital (reference: P111002-IDRCB2008-AO1465-50) in Paris, France. All participants provided written informed consent prior to inclusion. All procedures involving these participants were performed in accordance with the ethical standards of the institutional and national committee, with relevant French regulations and guidelines, and with the 1964 Helsinki declaration and its later amendments or comparable standards.

### Sample preparation

Lymphoblastoid cell lines (LCLs), established following standard procedures (Neitzel 1986), were cultured in RPMI-1640 medium containing 2 mM of L-glutamine and supplemented with 10% fetal bovine serum and 1% penicillin/streptomycin (Life Technologies, France) in a 5% CO_2_ humidified incubator at 37 °C. LCLs were seeded at 4 × 10^5^ cells/ml. After 4 days cells were harvested for RNA isolation. Total RNA was extracted from 5 × 10^6^ cells pellets using the miRNeasy Mini Kit according to the manufacturer’s protocol (QIAGEN, France) and quantified with a NanoDrop One spectrophotometer (ThermoFisher Scientific, France). Total RNA with a concentration > 50 ng/µl and absorbance ratios at 260/280 nm between 1.8 and 2.1, according to the MIQE guidelines, were stored at −80 °C until processing.

### RT-qPCR measures

1 µg of total RNA was reverse transcribed, in a final volume of 50 µl, using the iScript Reverse Transcription Supermix following the manufacturer’s protocol (Bio-Rad laboratories, France) and incubated5 min at 25 °C, 20 min at 46 °C followed by 1 min at 95 °C. After reverse transcription, cDNA were stored at −20 °C. For reference genes selection we used the pre-plated 384 wells reference panel with 30 commonly used reference genes in human studies (Bio-Rad laboratories, France). SsoAvanced Universal SYBR Green Supermix (Bio-Rad laboratories, France) was used for amplification following the manufacturer’s instructions. The thermal cycling conditions were as follow: polymerase activation 95 °C 2 min, denaturation 95 °C 5 s, annealing extension 60 °C 30 s for 40 cycles. To verify the specificity of PCR products, a melting curve analysis step was performed: 65–95 °C with a 0.5 °C increment and 2–5 s/step. PCR reactions were carried out in triplicate using a 7900HT instrument (Applied Biosystem). The data were analyzed using the SDS 2.4 software (Applied Biosystem). Baseline correction and threshold setting were performed using the automatic calculation offered by the same software. To eliminate atypical values, Cq values for a given sample were averaged; when their standard deviation was higher than 0.5 cycle the value showing the greatest deviation from the average was eliminated. In addition, when at least one of the replicates were detected after 32 cycles or not detected at all, the triplicate was labelled as “failed”.

### Data analysis

All analyses were done on the Cq values, in the log scale using R version 3.5.1 and additional package SARP.compo version 0.0.9^2^. Algorithms for geNorm and NormFinder were rewrote in R and checked against the original software and their usual implementation in R. Results, expressed as mean Cq of triplicates, are available as an example dataset in the SARP.compo package, available on the Comprehensive R Archive network^17^. Raw, individual Cq values are available as Supplementary Material.

#### Equivalence tests procedure

The equivalence region was defined as [−Δ, +Δ] = [−0.5, +0.5], in the Cq scale. This means that any change lower than a 1.414 fold change (assuming a 100% efficiency of the amplification) was considered as biologically irrelevant. Confidence intervals were built assuming a Gaussian distribution of Cq values, with equal variance.

Based on this equivalence region, all pairwise ratios were tested and the graph built. To ensure a nominal 0.05 level for the rejection of the null hypothesis “all genes behave differently”, the individual equivalence test rejection level was adjusted by simulation, according to the method previously published^2^. 10,000 simulations were done.

To check the influence of the equivalence region choice, the same approach was also used varying Δ. Especially, alternative values for Δ were selected according to the observed analytical and inter-individuals variabilities of the genes.

## Supplementary information

How to use the method?

How to use the method? (R code)

Supplementary Dataset 1

Mathematical model underlying gene ranking or selection

## Data Availability

The data of the thirteen candidate reference gene is included in the SARP.compo package, starting with version 0.1.0. An example on how to build a graph (for Δ = 0.5, a cutoff of *p* = 0.15 and 10 of the 30 genes) is given in the documentation page of the equiv.fpc function of the same package; this example can be adapted to reproduce the Fig. 3 of the paper by including the 30 genes and setting *p* = 0.30. This package is freely available on the CRAN website. The data and the R source code for the tutorial given as Supplementary Material are also available as Supplementary Material.

## References

[1] Bustin SA (2009). The MIQE guidelines: minimum information for publication of quantitative real-time PCR experiments. Clin. Chem..

[2] Curis E (2019). Determination of sets of covariating gene expression using graph analysis on pairwise expression ratios. Bioinforma. Oxf. Engl..

[3] Vandesompele, J. *et al*. Accurate normalization of real-time quantitative RT-PCR data by geometric averaging of multiple internal control genes. *Genome Biol*. **3**, RESEARCH0034 (2002). 10.1186/gb-2002-3-7-research0034 PMC126239 12184808

[4] Andersen CL, Jensen JL, Ørntoft TF (2004). Normalization of real-time quantitative reverse transcription-PCR data: a model-based variance estimation approach to identify genes suited for normalization, applied to bladder and colon cancer data sets. Cancer Res..

[5] De Spiegelaere W (2015). Reference gene validation for RT-qPCR, a note on different available software packages. PloS One.

[6] Zhang Q (2018). Selection and Validation of Reference Genes for RT-PCR Expression Analysis of Candidate Genes Involved in Morphine-Induced Conditioned Place Preference Mice. J. Mol. Neurosci. MN.

[7] Palve V (2018). A minimal set of internal control genes for gene expression studies in head and neck squamous cell carcinoma. PeerJ.

[8] Zhang K (2019). Selection and validation of reference genes for target gene analysis with quantitative real-time PCR in the leaves and roots of Carex rigescens under abiotic stress. Ecotoxicol. Environ. Saf..

[9] Panahi, Y. *et al*. Selection of Suitable Reference Genes for Analysis of Salivary Transcriptome in Non-Syndromic Autistic Male Children. *Int. J. Mol. Sci*. **17** (2016). 10.3390/ijms17101711 PMC5085743 27754318

[10] Molina CE (2018). Identification of optimal reference genes for transcriptomic analyses in normal and diseased human heart. Cardiovasc. Res..

[11] Haller F (2004). Equivalence test in quantitative reverse transcription polymerase chain reaction: confirmation of reference genes suitable for normalization. Anal. Biochem..

[12] Lakens D (2017). Equivalence Tests: A Practical Primer for t Tests, Correlations, and Meta-Analyses. Soc. Psychol. Personal. Sci..

[13] Wellek S, Blettner M (2012). Establishing Equivalence or Non-Inferiority in. Clinical Trials. Dtsch. Ärztebl. Int..

[14] Lu D (2015). International Guidelines for Bioequivalence of Locally Acting Orally Inhaled Drug Products: Similarities and Differences. AAPS J..

[15] Li Z, Fang L, Jiang W, Kim M-J, Zhao L (2017). Risk-Based Bioequivalence Recommendations for Antiepileptic Drugs. Curr. Neurol. Neurosci. Rep..

[16] Walker E, Nowacki AS (2011). Understanding Equivalence and Noninferiority Testing. J. Gen. Intern. Med..

[17] Curis, E. *SARP.compo: Network-Based Interpretation of Changes in Compositional Data*. (2019).

[18] Etain B (2014). Association between circadian genes, bipolar disorders and chronotypes. Chronobiol. Int..

