# Supplementary material for: Selecting reference genes in RT-qPCR based on equivalence tests: a network based approach
Source: Sci Rep. 2019 Nov 7;9:16231. doi: 10.1038/s41598-019-52217-2 (PMC6838083; doi:10.1038/s41598-019-52217-2)
Supplement: Supplementary file 1 — How to use the method? [file 41598_2019_52217_MOESM1_ESM.pdf]

Selecting reference genes in RT-qPCR based on equivalence tests: a network based approach

Emmanuel CURIS*[1, 2, 3], Calypso NEPOST[1], Diane GRILLAULT-LAROCHE [1,4], Cindie COURTIN[1], Jean-Louis LAPLANCHE[1], Bruno ETAIN [1, 4, 5], Cynthia MARIE-CLAIRE[1]

1. UMR-S 1144, INSERM Optimisation thérapeutique en neuropsychopharmacologie – université Paris Descartes – université Paris Diderot

2. Laboratoire de biomathématiques, EA 7537 BioSTM, faculté de pharmacie de Paris, université Paris Descartes, Paris, France

3. Service de biostatistiques et d'informatique médicale, hôpital Saint-Louis, AP-HP, Paris, France

4. AP-HP, GH Saint-Louis – Lariboisière – F. Widal, Pôle de psychiatrie et de médecine addictologique, 75475 Paris cedex 10, France

5. Fondation Fondamental, Créteil, France

* : corresponding author, emmanuel.curis@parisdescartes.fr

SUPPLEMENTARY MATERIAL: case study of reference gene selection – a tutorial on method usage

To illustrate further the method, and to help the readers to apply the method in their current practice, we explain in detail in this document a case study, in conditions closest to usual practice (testing 30 candidate reference genes being quite unusual).

Experiment description

Data used in this tutorial are data coming from RT-qPCR analysis of gene expression levels between two samples of patients, described below as patients of type A and B. The exact nature of these two types is here irrelevant. The genes quantified included 5 potential reference genes: *ACTB*, *GAPDH*, *HPRT1*, *RPLP0* and *SDHA*. There was 14 patients of type A and 19 of type B.

RT-qPCR experiments

Total RNA was extracted from lymphoblastoid cell lines (LCLs) of the patients. LCLs were cultured in RPMI-1640 medium containing 2 mM of L-glutamine and supplemented with 10 % fetal bovine serum and 1 % penicillin/streptomycin (Life Technologies, France) in a 5 % CO_2 humidified incubator at 37 °C. LCLs were seeded at 2×10^5 cells/mL. After 4 days, cells were harvested for RNA isolation. Total RNA was extracted from 5×10^6 cells pellets using the *miRNeasy Mini Kit* according to the manufacturer's protocol (QIAGEN, France) and quantified with a *NanoDrop One* spectrophotometer (ThermoFisher Scientific, France). Total RNA were stored at −80 °C until processing.

1 µg of total RNA was reverse transcribed, using the *iScript Reverse Transcription Supermix* following the manufacturer's protocol (Bio-Rad laboratories, France). After reverse transcription, cDNA were aliquoted and stored at −20 °C. Custom designed 384 wells *Prime PCR* plates were used. Five potential reference genes (Bio-Rad laboratories, France) were tested. *SsoAvanced Universal SYBR® Green Supermix* (Bio-Rad laboratories, France) was used for amplification following the manufacturer's instructions. The amplification was performed on a *7900HT* instrument (Thermofischer). The specificity of PCR products was verified using a melting curve analysis step. Assays were carried out in duplicate. Baseline correction and threshold setting were performed using automatic calculation.

Raw results

Results of the RT-qPCR quantification for the 5 candidate genes are summarized in the Table below. All results are expressed on the Cq scale. To eliminate atypical values, Cq values for a given sample were averaged. In addition, when at least one of the replicates was detected after 32 cycles or not detected at all, the duplicate was labelled as "not detected". A Gaussian (normal) distribution of the Cq values was assumed. Of note, two genes (*ACTB* and *RPLP0*) have quite higher standard deviation than the three others, making them less acceptable reference genes.

Gene	Summary statistics (Cq scale, n = 33)				
	min	*median*	*mean*	*max*	*standard deviation*
ACTB	13.34	19.83	18.79	21.95	2.24
GAPDH	11.83	17.11	16.58	19.00	1.70
HPRT1	18.59	22.81	22.50	24.84	1.38
RPLP0	13.64	15.68	16.88	35.94	3.85
SDHA	17.35	21.54	21.27	23.34	1.51

Equivalence region determination

The choice of Δ is the first key step to apply the method. Selecting Δ too high will allow large difference in expression changes of reference genes between conditions A and B, hence normalizing using these genes will lead to results that are difficult to interpret. On the contrary, imposing a too stringent Δ will make the method fail, even for perfectly equivalent genes, simply because the sample size does not allow a narrow enough confidence interval to fit in the [−Δ, + Δ] equivalence region.

For the first criterion, the lower Δ is the better. We think that Δ = 1 should be the maximum relevant value; it corresponds, for a 100 % efficacy of the amplification, to doubling the amount of one gene compared to the other in condition B compared to condition A.

For the second criterion, we note that (assuming a Gaussian distribution) the confidence interval is of width 2 t s, where t is the 0.95-quantile of a Student law with (14 + 19 − 2) = 31 degrees of freedom (for an equivalence test with a Type I error of 5 %, the confidence interval should have a 90 % confidence); that is, $t \approx 1.696$; and s is the observed standard deviation of the difference. Since we are interested in changes of the ratio between genes i and j between conditions A and B, the observed mean difference in the Cq scale is given by $(m_{j,\mathrm{B}} - m_{i,\mathrm{B}}) - (m_{j,\mathrm{A}} - m_{i,\mathrm{A}})$ where $m_{i,c}$ is the mean expression [in Cq scale] of gene i in condition c. Hence, its observed standard deviation is given by

$$s = \sqrt{\frac{s_{j,B}^2}{n_B} + \frac{s_{i,B}^2}{n_B} + \frac{s_{j,A}^2}{n_A} + \frac{s_{i,A}^2}{n_A}}$$

If one assumes equal standard deviation for all genes, a reasonable hypothesis if genes are equally good reference genes, and replace the observed standard deviation by 1.53 (which is the average standard deviation observed for the three candidate reference genes with the lowest standard deviation), that gives s ≈ 0.762. Hence, the expected width of the confidence interval is around 2×1.696×1.064 = 2.58 Cq: this value being much higher than Δ = 1 means that using lower values for Δ is here irrelevant.

Consequently, the equivalence region was set as Δ = 1 Cq, that is [−1, +1], for this experiment.

Determining the *p*-value cut-off

The second key step of the method is to define which criterion will be used, on the graph, to decide that two genes are equivalent − hence potential reference genes −, and to select a p-value cut-off for each individual equivalence test, so that the Type I error (α) of having this criterion fulfilled in the obtained graph, when genes are in reality not equivalent, is controlled (typically, α ≤ 5 %).

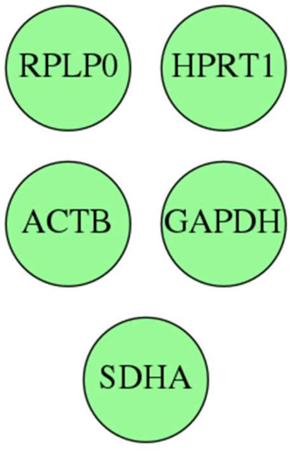

Since the total number of candidate genes (nodes of the graph) is low, we choose in this tutorial the simplest criterion: two genes are said equivalent if they are connected in the graph. Hence, under the null hypothesis that all genes have different expression changes between A and B, the graph should be fully disconnected, with 5 nodes and no edge (figure on the right).

To select the p-value cutoff and control the Type I error, simulations under the null hypothesis must be achieved. The corresponding R source code is given as another supplementary file. To be on the frontier between the two hypotheses, the difference of expression between conditions A and B were of 0, +1, +2, +3, and +4 Cq for all genes, leading to the simulation conditions summarized in the table below. Using these conditions, and assuming a Gaussian distribution of expectation value μ and of standard deviation σ, Cq values were simulated for the 14 patients in condition A and the 19 patients in condition B. These values were then analyzed doing all pairwise equivalence tests, and the corresponding graph built using different p-value cutoffs. If the graph presents a least one edge, equivalence of at least two genes is (wrongly) concluded, the null hypothesis is (wrongly) rejected.

Gene	Condition A		Condition B	
	μ (Cq)	σ (Cq)	μ (Cq)	σ (Cq)
ACTB	15	1.5	15	1.5
GAPDH	15	1.5	15 + 1 = 16	1.5
HPRT1	15	1.5	15 + 2 = 17	1.5
RPLP0	15	1.5	15 + 3 = 18	1.5
SDHA	15	1.5	15 + 4 = 19	1.5

This simulation was done 10'000 times. Figure below shows the evolution of the Type I error with the *p*-value cutoff. The *p*-value cutoff was defined as the cutoff for which 5 % of the simulation gave a graph with at least one edge (that is, one rejects the null hypothesis incorrectly). This gives a *p*-value cutoff of approximately 0.132.

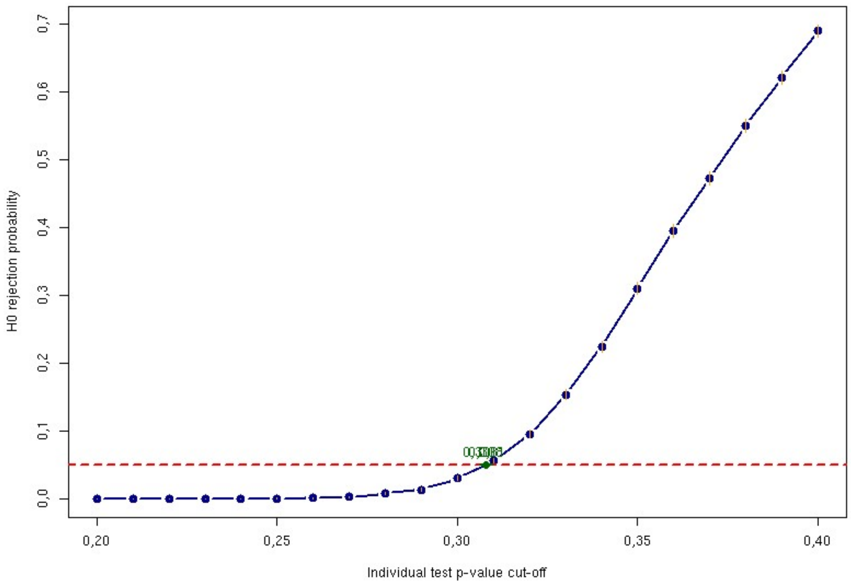

Application of the method

Once the cutoff is selected, the method is applied on experimental data: p-values for all pairwise equivalence tests are computed and the corresponding graph is built using the p-value cutoff defined previously. This leads to the graph on the right.

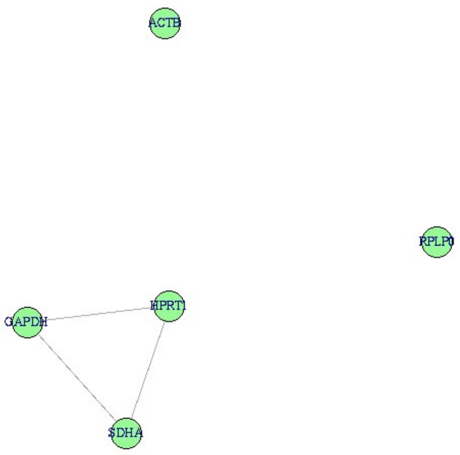

Clearly, this graph shows two isolated nodes, ACTB and RPLP0, and a maximal clique (completely connected graph) including the three other nodes. Hence, the three genes *ACTB*, *HPRT1* and *SDHA* can be said equivalent (based on the [−1, +1] equivalence region defined) and are good candidates to be reference genes. Of note, the two rejected genes are the one with the highest experimental standard deviation, for which a warning was previously issued based on these "high" standard deviations.

Comparison to other methods

The figure below shows the ranking of these genes by the geNorm (left) and NormFinder (right) methods. In this particular example, the two methods give the same ranking. Both of them detect the three selected genes as the "best" reference genes. However, there is no criterion to select only these three genes, or all of them: the "best" one or the "two best" ones could be also selected. This is especially true for the geNorm method, for which the progression of the instability index is regular, whereas in the NormFinder ranking, the decrease of the rho index is higher for the first two genes.

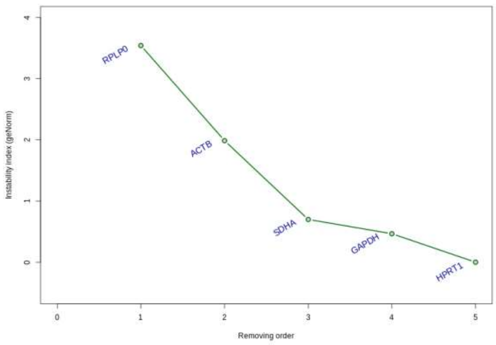
Instability index (geNorm)
Removing order
RPLP0
ACTB
SDHA
GAPDH
HPRT1

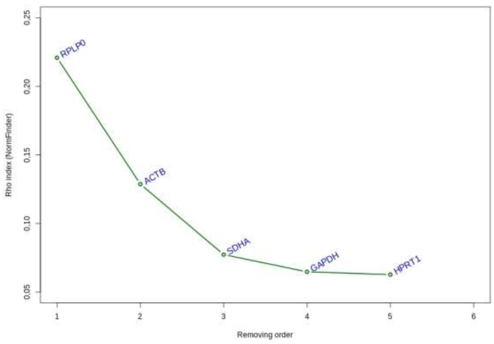
Rho index (NormFinder)
Removing order
RPLP0
ACTB
SDHA
GAPDH
HPRT1
