# Supplementary material for: Selecting reference genes in RT-qPCR based on equivalence tests: a network based approach
Source: Sci Rep. 2019 Nov 7;9:16231. doi: 10.1038/s41598-019-52217-2 (PMC6838083; doi:10.1038/s41598-019-52217-2)
Supplement: Supplementary file 4 — Mathematical model underlying gene ranking or selection [file 41598_2019_52217_MOESM4_ESM.pdf]

Selecting reference genes in RT-qPCR based on equivalence tests : a network based approach

Emmanuel Curis, Calypso Nepost, Diane Grillault Laroche, Cindie Courtin, Jean-Louis Laplanche, Bruno Etain,Cynthia Marie-Claire

novembre 2018 — Variapsy / DBIM Saint-Louis / Laboratoire de biomathématiques, EA 7537

Supplementary : Mathematical model for gene expression, in reference genes context

Introduction

Model of individual RNA quantified amount

Let consider a set of K different RNAs, of which some will be quantified using qRT-PCR ; let $k = 1 \dots K$ the gene index. This quantification will be done for C conditions ; let $c = 1 \dots C$ the condition index. In each condition, experiment will be done on n_c samples ; let $i = 1 \dots n_c$ be the sample index. With these notations, the amount of RNA k in the sample i for condition c is given by a random variable, $\mathrm{X}_{k,c,i}$.

For sake of simplicity, expressions will be given in the case of two conditions, $C = 2$, and the first condition will be considered as the reference one — they can easily be extended to more than two conditions. In the two-conditions case, one can write

$$\log \mathrm{X}_{k,c,i} = \mu_k + \delta_k \, \mathbb{1}_{c=2} + \varepsilon_{k,c,i}$$

where μ_k is (in log) the average expression level of RNA k in condition 1, δ_k the average change in expression level of this RNA in condition 2, $\mathbb{1}_{c=2}$ a function that equals 1 if the measure is done in condition 2, 0 otherwise and $\varepsilon_{k,c,i}$ an centered random variable ($\mathbb{E}(\varepsilon_{k,c,i}) = 0$), of variance $\sigma^2_{k,c}$, which includes the between-sample variation and the intra-sample variation.

Control genes, also called reference genes or housekeeping genes, are genes that are assumed to have similar expression levels between conditions, that is $\delta_k = 0$. In addition, best control genes will be the one with the smallest variances, that is both $\sigma^2_{k,1}$ and $\sigma^2_{k,2}$ minimal. All methods to select control genes will try to select genes that satisfy these conditions.

The experimental setup raises additional difficulties. First, After the retro-transcription step, the amount of the corresponding cDNA is $\mathrm{X}^{\mathrm{c}}_{k,c,i} = \rho_k \, \mathrm{X}_{k,c,i}$, where ρ_k is the retro-transcription yield for RNA k. Because of the extraction of a predefined amount of *total* DNA after retro-transcription, however, the quantified amount is

$$\mathrm{X}^*_{k,c,i} = \frac{\mathrm{X}^{\mathrm{c}}_{k,c,i}}{\sum_{k=1}^{K} \mathrm{X}^{\mathrm{c}}_{k,c,i}} = \frac{\rho_k \, \mathrm{X}_{k,c,i}}{\sum_{k=1}^{K} \rho_k \, \mathrm{X}_{k,c,i}}.$$

The result of the quantification is finally $\mathrm{Y}^*_{k,c,i} = \lambda_k \mathrm{X}^*_{k,c,i}$, where λ_k is the, unknown, proportionnality constant between the obtained result (typically, C_q in the log scale) and the amount of the RNA of interest in the well. Finally, the model for the available data is

$$\mathrm{Y}_{k,c,i} = \lambda_k \frac{\rho_k \, \mathrm{X}_{k,c,i}}{\sum_{k=1}^{K} \rho_k \, \mathrm{X}_{k,c,i}} = \lambda_k \frac{\rho_k \, \mathrm{e}^{\mu_k + \delta_k \mathbb{1}_{c=2} + \varepsilon_{k,c,i}}}{\sum_{k=1}^{K} \rho_k \, \mathrm{e}^{\mu_k + \delta_k \mathbb{1}_{c=2} + \varepsilon_{k,c,i}}}.$$

Both ρ_k and λ_k are assumed to depend only on the RNA, but neither on the experimental condition, nor on the patient ; unavoidable variations of these values between samples will be included in the error term of the model.

Expression of the ratios

We define $R_{k,k',c,i}$ the ratio of the measured expressions of genes k and k', in a given sample. We have

$$R_{k,k',c,i} = \frac{\mathrm{Y}_{k',c,i}}{\mathrm{Y}_{k',c,i}} = \frac{\lambda'_k}{\lambda_k} \frac{\rho'_k}{\rho_k} \frac{X_{k',c,i}}{X_{k,c,i}} = \frac{\lambda_{k'}}{\lambda_k} \frac{\rho_{k'}}{\rho_k} \exp\left(\Delta_{k,k'}\mu + \Delta_{k,k'}\delta \mathbb{1}_{c=2} + \Delta_{k,k'}\varepsilon_{c,i}\right)$$

where $\Delta_{k,k'}\mu = \mu'_k - \mu_k$ and so on.

We have $\mathbb{E}(\log R_{k,k',c,i}) = \log \frac{\lambda'_k}{\lambda_k} \frac{\rho'_k}{\rho_k} + \Delta_{k,k'}\mu + \Delta_{k,k'}\delta \mathbb{1}_{c=2}$.

We have $\mathbb{V}(\log R_{k,k',c,i}) = \sigma^2_{k,k',c} = \mathbb{V}(\Delta_{k,k'}\varepsilon_{c,i}) = \sigma^2_{k,c} + \sigma^2_{k',c} - 2\,\rho_{k,k',c}\,\sigma_{k,c}\,\sigma_{k',c}$, with $\rho_{k,k',c}$ the linear correlation coefficient between values for genes k and k' in condition c.

1 All ratios of ratios method

For each pair of genes, k and k', we consider the eventual change in their ratio, $R_{k,k',c,i}$, between the two conditions. In case of such a change, the ratio of these ratios is different from 1 — that is, $\frac{R_{k,k',2,i}}{R_{k,k',1,i}} \neq 1$. We have indeed

$$\mathbb{E}\left(\log \frac{R_{k,k',2,i}}{R_{k,k',1,i}}\right) = \mathbb{E}(\log R_{k,k',2,i} - \log R_{k,k',1,i}) = \Delta_{k,k'}\delta.$$

Consequently, one can estimate $\Delta_{k,k'}\delta$, which is the difference of changes of expression of genes k and k' between the two conditions. If both genes are control genes, then $\Delta_{k,k'}\delta = 0$, so identification of control genes is the identification of pairs of genes for which $\Delta_{k,k'}\delta = 0$.

In general, samples are different between the two conditions, hence one cannot access this ratio individual values, and its average will be estimated, in the log-scale, by the difference of the averages of the values in each condition ("two independant samples comparison if mean").

Assuming a Gaussian distribution for $\varepsilon_{k,c,i}$, the test of $\Delta_{k,k'}\delta = 0$ can be done for all pairs of genes, either using an usual Student's T-test (and if the test of $R_{k,k',c,j}$ is non-significant, the pairs of genes k and k' will be declared as candidate control genes) or an equivalence test of $\Delta_{k,k'}\delta$ to 0 (and if the test of $R_{k,k',c,j}$ is non-significant, the pairs of genes k and k' will be declared as candidate control genes).

For an equivalence test, the test criterion is based on the confidence interval of the difference of the means, hence will be given here, assuming a Gaussian distribution and denoting arithmetic means on the samples by overline, by

$$\left[\overline{\log R_{k,k',2,i}} - \overline{\log R_{k,k',1,i}} + t_{\frac{\alpha}{2},\nu} S_{k,k'} ; \overline{\log R_{k,k',2,i}} - \overline{\log R_{k,k',1,i}} + t_{1-\frac{\alpha}{2},\nu} S_{k,k'}\right]$$

where $S_{k,k'}$ is the estimation of the variance of this difference; in the independant samples cases, we have $S^2_{k,k'} = \frac{S^2_{k,k',1}}{n_1} + \frac{S^2_{k,k',2}}{n_2}$ and

$$S^2_{k,k',c} = \frac{1}{n_c - 1} \sum_{i=1}^{n_c} \left(\log R_{k,k',c,i} - \overline{\log R_{k,k',c,i}}\right)^2.$$

The expectation of this confidence interval will be

$$\left[\delta_{k'} - \delta_k + t_{\frac{\alpha}{2},\nu} \sqrt{\frac{\sigma^2_{k,k',1}}{n_1} + \frac{\sigma^2_{k,k',2}}{n_1}} ; \delta_{k'} - \delta_k + t_{1-\frac{\alpha}{2},\nu} \sqrt{\frac{\sigma^2_{k,k',1}}{n_1} + \frac{\sigma^2_{k,k',2}}{n_1}}\right]$$

which includes the average change of expression between the two conditions, but also correlations between genes and their variabilities, through the $\sigma^2_{k,k',c}$ terms.

Finally, all possible couples of genes are tests and, according to the methodology described in [Curis et al., *BioInformatics*, 2019], a graph of genes is built and analyzed.

2 The geNorm method

The original geNorm model does not specify an explicit model for RNA amounts. It is a descriptive approach in three steps : first, compute all pairwise ratios, second estimate the standard deviation of these ratios for all pairs of RNAs and third, for each RNA, average the standard deviation of all ratios involving it. The second and third step are performed on the binary log scale.

The first step is equivalent to the first one in the previous methd, and with the suggested expression model, it corresponds to computing the $R_{k,k',c,i}$ values defined above. Using the Vandesompele et al. notations, with indices translated to our conventions, that gives

$$A_{k,k'} = \{\log_2 R_{k,k',c,i}\}_{\substack{c=1,2\\ i=1\dots n}} = \frac{1}{\log 2} \left\{\log \frac{\lambda_{k'}\,\rho_{k'}}{\lambda_k\,\rho_k} + \Delta_{k,k'}\mu + \Delta_{k,k'}\delta\,\mathbb{1}_{c=2} + \Delta_{k,k'}\varepsilon_{c,i}\right\}_{\substack{c=1,2\\ i=1\dots n}}$$

Let

$$\overline{\overline{A_{k,k'}}} = \frac{1}{n_1 + n_2} \sum_{c=1}^{2} \sum_{i=1}^{n_c} \log_2 R_{k,k',c,i} = \frac{1}{\log 2} \left(\log \frac{\lambda_{k'}\,\rho_{k'}}{\lambda_k\,\rho_k} + \Delta_{k,k'}\mu + \frac{n_2}{n_1 + n_2} \Delta_{k,k'}\delta + \frac{1}{n_1 + n_2} \sum_{c=1}^{2} \sum_{i=1}^{n_c} \Delta_{k,k'}\varepsilon_{c,i}\right)$$

be the average of the whole $A_{k,k'}$ set – that is, the average of all available ratios for the (k, k') pair of RNA, the "grand mean".

Let

$$\overline{A^c_{k,k'}} = \frac{1}{n_c} \sum_{i=1}^{n_c} \log_2 R_{k,k',c,i} = \frac{1}{\log 2} \left(\log \frac{\lambda_{k'}\,\rho_{k'}}{\lambda_k\,\rho_k} + \Delta_{k,k'}\mu + \Delta_{k,k'}\delta\,\mathbb{1}_{c=2} + \frac{1}{n_c} \sum_{i=1}^{n_c} \Delta_{k,k'}\varepsilon_{c,i}\right)$$

be the average of these ratios, limited to the condition c. We have

$$V_{k,k'}^2 = \frac{1}{n_1+n_2-1}\sum_{c=1}^{2}\sum_{i=1}^{n_c}\left(\log_2 R_{k,k',c,i} - \overline{\overline{A_{k,k'}}}\right)^2$$

Application of the usual one-way analysis of variance sum of square decomposition gives

$$\begin{aligned}(n_1+n_2-1)V_{k,k'}^2 &= \sum_{c=1}^{2}\sum_{i=1}^{n_c}\left(\log_2 R_{k,k',c,i} - \overline{A_{k,k'}^c}\right)^2 + \sum_{c=1}^{2}\sum_{i=1}^{n_c}\left(\overline{A_{k,k'}^c} - \overline{\overline{A_{k,k'}}}\right)^2 \\ &= \frac{1}{\log 2}\left[(n_1-1)\hat{\sigma}_{k,k',1}^2 + (n_2-1)\hat{\sigma}_{k,k',2}^2 + \frac{n_1\,n_2}{n_1+n_2}\left(\Delta_{k,k'}\delta + (\hat{\mu}_{k,k',2} - \hat{\mu}_{k,k',1})\right)^2\right]\end{aligned}$$

where $\hat{\mu}_{k,k',c} = \frac{1}{n_c}\sum_{i=1}^{n_c}\Delta_{k,k'}\varepsilon_{c,i}$ and $\hat{\sigma}_{k,k',c}^2 = \frac{1}{n_c-1}\sum_{i=1}^{n_c}\left(\Delta_{k,k'}\varepsilon_{c,i} - \frac{1}{n_c}\sum_{i=1}^{n_c}\Delta_{k,k'}\varepsilon_{c,i}\right)^2$ is the unbiased estimator of $\sigma_{k,k'}^2$, the (k,k') ratio variance in condition c, in the log scale.

Considering only the expectation of this expression, with have

$$\begin{aligned}\mathbb{E}(V_{k,k'}^2) &= \frac{1}{\log 2}\left[\frac{(n_1-1)\sigma_{k,k',1}^2 + (n_2-1)\sigma_{k,k',2}^2}{n_1+n_2-1} + \frac{n_1\,n_2((\Delta_{k,k'}\delta)^2 + \sigma_{k,k',1}^2 + \sigma_{k,k',2}^2)}{(n_1+n_2)(n_1+n_2-1)}\right] \\ &= \frac{1}{\log 2}\frac{1}{n_1+n_2-1}\left[\left(n_1 - 1 + \frac{n_1\,n_2}{n_1+n_2}\right)\sigma_{k,k',1}^2 + \left(n_2 - 1 + \frac{n_1\,n_2}{n_1+n_2}\right)\sigma_{k,k',2}^2 + \frac{n_1\,n_2}{n_1+n_2}(\Delta_{k,k'}\delta)^2\right]\end{aligned}$$

To have lighter notations, let $\overline{n} = \frac{n_1\,n_2}{n_1+n_2}$ and $A_c = n_c - 1 + \overline{n}$: the expectation then rewrites as

$$\mathbb{E}(V_{k,k'}^2) = \frac{1}{\log 2}\frac{1}{n_1+n_2-1}\left(A_1\,\sigma_{k,k',1}^2 + A_2\,\sigma_{k,k',2}^2 + \overline{n}(\Delta_{k,k'}\delta)^2\right)$$

The last step computes, for a given RNA (k), the average of the $V_{k,k'}$ for all $k' \neq k$, which gives

$$M_k = \frac{1}{K^*-1}\sum_{k'=1}^{K^*} V_{k,k'}$$

where K^* is the number of considered genes amongst the K. The genes are then sorted according to this value.

Understanding the exact origin of this sorting order, even considering ideal values instead of the random variables, is not straightforward, partly because of the use of the standard deviation that introduces a square root, and partly because each value involves the relations between a gene and all other genes.

Nevertheless, if one consider the special case of a set of candidate reference genes, with one gene, let's say $k=1$, that is not a reference and all other genes are indeed, this means that $\Delta_{k,k'}\delta = 0$ except for $k=1$ where $\Delta_{k,k'}\delta = d$. In that case, and also assuming that variances are the same for all genes and all conditions, then for all reference genes $(k>1)$ the $\Delta\delta_{k,k'}^2$ term will be present in only $V_{k,1}$, which mean it will have influence roughly of $\frac{d}{K^*-1}$, whereas for the "outlier" gene, it will be present in all $V_{1,k'}$ terms, which mean it will have influence roughly of d — all other parts beeing equal. In other words, the method will detect a gene for which expression changes among a set of non-varying genes, and the more non-varying genes, the easiest is the detection by ranking the M_k values.

For more complex models, using the expressions above can give the expected classification of the genes according to the method.

To further explore qualitatively the characteristics of the method, we consider instead the measure

$$M_k^* = \frac{1}{K^*-1}\sum_{k'=1}^{K^*} V_{k,k'}^2.$$

Despite there is no simple relation between M_k and M_k^*, factors changing M_k^* should change M_k in the same way, allowing to understand what drives the final ranking of the genes. We have

$$\mathbb{E}(M_k^*) = \frac{1}{K^*-1}\sum_{k'=1}^{K^*}\mathbb{E}(V_{k,k'}^2) = \frac{1}{\log 2}\frac{1}{n_1+n_2-1}\left[\frac{1}{K^*-1}\sum_{k'=1}^{K^*}\left(A_1\,\sigma_{k,k',1}^2 + A_2\,\sigma_{k,k',2}^2 + \overline{n}(\Delta_{k,k'}\delta)^2\right)\right]$$

By replacing $\sigma_{k,k',c}^2 = \sigma_{k,c}^2 - 2\,\rho_{k,k',c}\,\sigma_{k,c}\,\sigma_{k',c} + \sigma_{k',c}^2$ and $\Delta_{k,k'}\delta = \delta_k' - \delta_k$ by their expressions, we then have, after grouping terms,

$$\mathbb{E}(M_k^*) = \frac{1}{\log 2}\frac{1}{n_1+n_2-1}\left[A_1\,\sigma_{k,1}^2 + A_2\,\sigma_{k,2}^2 + \overline{n}\,\delta_k^2 + \frac{1}{K^*-1}\left(\begin{array}{l} A_1\sum_{k'=1}^{K^*}\sigma_{k',1}^2 + A_2\sum_{k'=1}^{K^*}\sigma_{k',2}^2 \\ -\,2\,A_1\,\sigma_{k,1}\sum_{k'=1}^{K^*}\rho_{k,k',1}\,\sigma_{k',1} - 2\,A_2\,\sigma_{k,2}\sum_{k'=1}^{K^*}\rho_{k,k',2}\,\sigma_{k',2} \\ +\,\overline{n}\sum_{k'=1}^{K^*}\delta_{k'}^2 - 2\overline{n}\sum_{k'=1}^{K^*}\delta_k\,\delta_{k'}\end{array}\right)\right.$$

This expression mainly contains two parts, the first part that describes the variability of the gene k and the second part is an average of what happens for all other genes. This second part is all the more negligible, for the ranking, than the number of genes is large, because it basically becomes the same for all genes. Hence, ranking is mainly based on the $A_1\,\sigma_{k,1}^2 + A_2\,\sigma_{k,2}^2 + \overline{n}\,\delta_k^2$ part. Genes with high variations, either between patients ($\sigma_{k,1}^2$ and $\sigma_{k,2}^2$) or, in average, between conditions δ_k^2 will have high M_k^* values, hence high M_k values, and will be excluded first.

Of note, in the usual algorithm implementation, the M_k values are reevaluated after each candidate gene exclusion ; hence, the last term becomes more variable between genes. We can then suspect that ranking of the last genes may be more sensitive to correlations between genes and all other terms appearing in this factor.

3 The NormFinder method

The original paper presenting this method does not explicitly makes the difference between $X_{k,c,i}$, the real amount in the original sample, and $Y_{k,c,i}$, the amount quantified in the sample. In other words, it assumes that $\forall k, \rho_k = \lambda_k = 1$ — an assumption that we will kept in this part.

The NormFinder ranking method is based on the following model, adapting the original paper notations to the one used here :

$$\log X_{k,c,i} = \alpha_{k,c} + \beta_{c,i} + \varepsilon_{k,c,i}^{\mathrm{NF}} \iff X_{k,c,i} = \mathrm{e}^{\alpha_{k,c}}\,\mathrm{e}^{\beta_{c,i}}\,\mathrm{e}^{\varepsilon_{k,c,i}^{\mathrm{NF}}}$$

where $\alpha_{k,c}$ is presented as the (average) expression level of gene k in condition c, $\beta_{c,i}$ as the total amount of RNAs in sample i of condition c (in log scale), and $\varepsilon_{k,c,i}^{\mathrm{NF}}$ is a random variable assumed to be centered, of variance ${\sigma_{k,c}^{\mathrm{NF}}}^2$ and all $\varepsilon_{k,c,i}^{\mathrm{NF}}$ are assumed to be independant. The model also assumes that $\alpha_{k,c} = \alpha_c + \varepsilon_{k,c}^{\alpha}$, where α_c is the average expression level of *all* genes in condition c and $\varepsilon_{k,c}^{\alpha}$ is a centered random variable, with $\mathbb{V}(\varepsilon_{k,c}^{\alpha}) = \gamma^2$.

An important remark should be made on this model : since $\mathrm{e}^{\beta_{c,i}}$ represents the amount of total RNA, one must have $X_{k,c,i} \leq \mathrm{e}^{\beta_{c,i}}$, that is $\mathrm{e}^{\alpha_{k,c}}\,\mathrm{e}^{\varepsilon_{k,c,i}^{\mathrm{NF}}} \leq 1$. In other words, since the second factor represents noise and should be either greater or less than one, the $\mathrm{e}^{\alpha_{k,c}}$ term represents the fraction that the RNA of interest represents in the total RNA and is constrained by $\mathrm{e}^{\alpha_{k,c}} < 1 \iff \alpha_{k,c} < 0$. This constraint is not consistent with the hypothesis of a Gaussian distribution for $\mathbb{V}(\varepsilon_{k,c}^{\alpha}) = \gamma^2$, assumed to develop the stability measure ; however, it might be acceptable or not, depending on the value of γ compared to α_c.

Let define $\theta_c = \frac{1}{n_c}\sum_{i=1}^{n_c}\beta_c$ the average total amount of RNA in condition c and $Z_{k,c} = \frac{1}{n_c}\sum_{i=1}\log X_{k,c,i}$ the average quantified amount of gene k in condition c, in log-scale. Let $\overline{\alpha_k} = \frac{\alpha_{k,1}+\alpha_{k,2}}{2}$ be the average expression level of gene k, all conditions taken together. The stability measure used to rank the gene is then defined, for two conditions, by $\rho_k^{\mathrm{NF}} = \frac{\rho_{k,1}^{\mathrm{NF}}+\rho_{k,2}^{\mathrm{NF}}}{2}$, with

$$\rho_{k,c}^{\mathrm{NF}} = \frac{\gamma^2\,|\alpha_{k,c} - \overline{\alpha_k}|}{\gamma^2 + \frac{{\sigma_{k,c}^{\mathrm{NF}}}^2}{n_c}} + \sqrt{\frac{{\sigma_{k,c}^{\mathrm{NF}}}^2}{n_c} + \frac{\gamma^2\,\frac{{\sigma_{k,c}^{\mathrm{NF}}}^2}{n_c}}{\gamma^2 + \frac{{\sigma_{k,c}^{\mathrm{NF}}}^2}{n_c}}}$$

which is the sum of the expectation (with sign removed) and the standard deviation of the deviation of the observed values and the average level in each conditions.

Linking this model with the one presented in introduction is not straightforward, first because they are developed in quite different perspective (absolute amounts in our case, relative amounts in the NormFinder case), and second because of different hypothesis. Basically, one should make correspond

$$\log \mathrm{X}_{k,c,i} = \mu_k + \delta_k\,\mathbb{1}_{c=2} + \varepsilon_{k,c,i} = \alpha_c + \beta_{c,i} + \varepsilon_{k,c}^{\alpha} + \varepsilon_{k,c,i}^{\mathrm{NF}}$$

with the additional equations

$$\mu_k + \delta_k\,\mathbb{1}_{c=2} = \alpha_c + \varepsilon_{k,c}^{\alpha} = \alpha_1 + \delta\alpha\,\mathbb{1}_{c=2} + \varepsilon_{k,c}^{\alpha} \quad \text{and} \quad \mathrm{e}^{\beta_{c,i}} = \sum_{k=1}^{K}\mathrm{X}_{k,c,i}$$

which gives $\varepsilon_{k,c,i} = \beta_{c,i} + \varepsilon_{k,c,i}^{\mathrm{NF}}$ and $\sigma_{k,c}^2 = \mathbb{V}(\beta_{c,i}) + {\sigma_{k,c}^{\mathrm{NF}}}^2$. Here lies a technical discrepancy between the two models : in our model, $\varepsilon_{k,c}^{\alpha}$ is fixed and $\beta_{c,i}$ is random, while in the NormFinder model, $\varepsilon_{k,c}^{\alpha}$ is random and $\beta_{c,i}$, while not specified, is also random. Besides, the $\mathbb{V}(\beta_{c,i})$ term is difficult to obtain, due to the non-linear relationship between $\beta_{c,i}$ and $X_{k,c,i}$; it depends on all the ${\sigma_{k,c}^{\mathrm{NF}}}^2$ values, but also the δ_k and μ_k.

Neglecting this, we can write

$$\alpha_0 = \frac{1}{K}\sum_{k=1}^{K}\mu_k \qquad \delta\alpha = \frac{1}{K}\sum_{k=1}^{K}\delta_k \qquad \gamma^2 = \frac{1}{K}\sum_{k=1}^{K}\left[(\mu_k - \alpha_0)^2 + \frac{1}{2}\,(\delta_k - \delta\alpha)^2\right]$$

which gives

$$|\alpha_{k,c} - \overline{\alpha_k}| = \frac{1}{2}\delta_k$$

and in turn, by letting $G_c = \frac{\sigma_{k,c}^{\mathrm{NF}}}{\gamma\sqrt{n_c}}$,

$$\rho_{k,c}^{\mathrm{NF}} = \frac{1}{2}\delta_k \left(\frac{1}{1+G_c^2}\right) + \frac{\sigma_{k,c}^{\mathrm{NF}}}{\sqrt{n_c}}\sqrt{1+\frac{1}{1+G_c^2}}$$

and finally

$$2\,\rho_k^{\mathrm{NF}} = \frac{1}{2}\delta_k \left(\frac{1}{1+G_1^2} + \frac{1}{1+G_2^2}\right) + \frac{\sigma_{k,1}^{\mathrm{NF}}}{\sqrt{n_1}}\sqrt{1+\frac{1}{1+G_1^2}} + \frac{\sigma_{k,2}^{\mathrm{NF}}}{\sqrt{n_2}}\sqrt{1+\frac{1}{1+G_2^2}}$$

By comparing this expression with the one obtained for the geNorm method, in the alternative M_k^* version, one can see that basically the same terms are used for ranking, however they are weighted with quite different coefficients. Hence, it is not surprising that ranking can lead to quite different results.
